# Isolation and characterization of *ex vivo* expanded mesenchymal stem cells obtained from a surgical patient

**DOI:** 10.3892/mmr.2014.2892

**Published:** 2014-11-06

**Authors:** JIA HUANG, HUIFAN SHA, GUAN WANG, GUOLIANG BAO, SHUN LU, QINGQUAN LUO, QIANG TAN

**Affiliations:** Department of Shanghai Lung Cancer Center, Shanghai Chest Hospital, School of Medicine, Shanghai Jiao Tong University, Shanghai 200030, P.R. China

**Keywords:** human bone marrow mesenchymal stem cells, isolation, *in vitro*, *in vivo*, morphological characteristics

## Abstract

The aim of the present study was to investigate the morphological characteristics and pluripotent differentiation potential of human bone marrow mesenchymal stem cells (hBMMSCs) *in vitro* and *in vivo*. Bone marrow cells were isolated from a rib fragment of an adult surgical patient, hBMMSCs were isolated based on plastic adherence and expanded *ex vivo* and phenotyping was performed. Pluripotent differentiation assays for adipogenesis, myogenesis and osteogenesis were conducted. Hematopoietic reconstruction of sublethally irradiated nude mice was performed by infusion of hBMMSCs. The gene expression profiles of early and late hBMMSCs were examined. The rate of CD31-positive cells was 31.1% in passage (P)4 hBMMSCs and 18.6% in P10 hBMMSCs. CD105 and CD106 were expressed in 99 and 95% of P25 hBMMSCs, respectively. Lipid droplets appeared at day 18 post induction. For osteogenesis, palpable masses were grossly observed from day 35 post inoculation of hBMMSCs. Hematoxylin and eosin staining further revealed chondrocytes and bone tissues. For myogenesis, at day six post subcutaneous inoculation, hBMMSCs differentiated into myocytes and were positive for myoglobin and MyoD1. In irradiated nude mice reconstituted by hBMMSCs, the white blood cell count briefly decreased following irradiation; however, it gradually recovered. In the irradiated nude mice reconstituted with hBMMSCs, CD45- and CD34-positive cells were detected 72 h post induction. Gene microarray analysis of P7 and P57 hBMMSCs demonstrated that 20 genes were upregulated >2 fold and 40 genes were downregulated >2 fold in P57 hBMMSCs. In conclusion, the isolated HBMMSCs possessed pluripotent differentiation potential and it was feasible and safe to use hBMMSCs within 30 passages.

## Introduction

Tissue-specific stem cells are present in numerous human tissues and have the potential to differentiate into one or multiple mature cell types. Tissue-specific stem cells represent an intermediate between pluripotent cells and fully committed mature cells, and are excellent models for studying cellular differentiation and tissue regeneration. Human bone marrow mesenchymal stem cells (hBMMSCs) are derived from bone marrow pluripotent stem cells and exhibit the homologous properties of stem cells, including self-renewal and the capacity to develop into multiple lineages (1–3). The isolation of hBMMSCs is simple due to their plastic adherence and ability to expand in culture (4–7). It has been reported that hBMMSCs have considerable therapeutic potential in several malignancies, including cardiovascular disease, cellular replacement therapy and tissue engineering (8–11).

hBMMSCs express numerous surface markers, including SH2, SH3, CD90, CD105 and CD106, but do not express hematopoietic stem cell markers, such as CD14, CD34 and CD45. hBMMSCs are stable and preserve their differentiation potential following long-term *ex vivo* culture and cryopreservation. hBMMSCs have been demonstrated to differentiate into myocytes, hepatocytes, osteoblasts, chondrocytes, fibroblasts and adipocytes (12), but also differentiate into hematopoietic cells and matrix cells, and under specific conditions, form myotubes and tendon (13–15). Furthermore, hBMMSC transplantation does not elicit immune rejection. Therefore, they may become a new source for tissue repair and regeneration. hBMMSCs are readily obtained from human bone marrow and expanded *ex vivo*, and are then transplanted back into patients following *in vitro* induction (16,17). Therefore, hBMMSCs have potential clinical utility for the use of stem cells for personalized medicine.

Even though hBMMSCs are efficiently recovered from bone marrow aspirates of healthy individuals and from those of patients suffering from severe diseases and injuries, they are an uncommon cell type in the bone marrow, accounting for <0.1% of nucleated cells in bone marrow aspirates. Therefore, expansion of hBMMSCs to obtain a stable source of hBMMSCs without losing the pluripotential of hBMMSCs is important. In the present study, hBMMSCs were obtained from a human subject, grown to passage (P)65 and the morphological characteristics as well as pluripotent differentiation potential of these hBMMSCs were investigated *in vitro* and *in vivo*.

## Materials and methods

### Isolation and expansion of bone marrow cells

The study was approved by the ethics committee of Shanghai Chest Hospital, (Shanghai, China). Bone marrow cells were isolated from the rib fragment from an adult surgical patient at the Department of Thoracic Surgery, Shanghai Chest Hospital, Shanghai Jiaotong University (Shanghai, China). Cells (1.5×10^8 )^ were plated in gelatin-coated T175 culture flasks and the mesenchymal cell population was isolated based on plastic adherence. The cells were cultured in Dulbecco’s modified Eagles’s medium (DMEM; high glucose; Gibco BRL, Grand Island, NY, USA) containing 10% fetal bovine serum (FBS), 50 μg/ml thymidine phosphorylase (ECGF), 50 *μ*g/ml heparin, penicillin 100 U/ml and streptomycin 100 mg/ml (Invitrogen Life Technologies, Carlsbad, CA, USA) at 37°C in a humidified atmosphere containing 5% CO_2_ for 24 h. For expansion of the mesenchymal cell population, the cells were incubated under standard culture conditions. On day three, non-adherent cells were removed by changing the medium. Thereafter, the medium was replaced twice a week. Following reaching confluence, the cells were trypsinized (0.25% v/v trypsin EDTA; Invitrogen Life Technologies), re-suspended in culture media and seeded at 1×10^3^ cells/cm^2^ [passage (P)1]. P10 to P57 cells were used in the present study. Prior to administration, the cells were resuspended in 250 μl serum-free DMEM. For the cellular proliferation assays, hBMMSCs were grown in 24-well plates at a density of 0.5×10^4^ cells/ml and were counted at different time intervals for plotting the growth curve and calculating the proliferation index.

### Transmission electron microscopy

For transmission electron microscopy, hBMMSCs (2×10^5^) were harvested and fixed with 2.5% electron microscopy-grade glutaraldehyde, post-fixed in 1% osmium tetroxide with 0.1% potassium ferricyanide, dehydrated in gradient ethanol (30–90%) and embedded in Epon. Ultrathin sections (65 nm) were cut, stained with 2% uranyl acetate and examined under a Hitachi H-600 transmission electron microscope (Hitachi, Tokyo, Japan).

### Flow cytometric analysis

To verify the presentation of hBMSC markers, phenotyping was routinely performed by flow cytometry on culture-expanded hBMMSCs. Cells of P2 were collected from confluent layers following incubation with 0.25% trypsin-EDTA for 5 min. The single-cell suspensions were washed with phosphate-buffered saline (PBS)/0.5% bovine serum albumin (BSA) prior to the staining. For direct staining, the cells were centrifuged (250 × g, 5 min) and re-suspended in cold PBS/0.5% BSA. A total of 1×10^6^ cells were incubated for 15 min on ice in the dark in cold PBS/0.5% BSA with titrated concentrations of *R*-phycoerythrin (PE)-conjugated monoclonal mouse anti-human CD29, -CD31, -CD38, -CD44, -CD59, -CD80, -CD86, -CD90, -CD105 and -CD106 antibodies or monoclonal fluorescein isothiocyanate (FITC) conjugated mouse anti-human CD9, -CD14 and -CD34 antibodies. The cells were then washed twice by centrifugation (250 × g, 5 min) and re-suspended with cold PBS/0.5% BSA prior to proceeding to flow cytometry. Dead cells and debris were stained with propidium iodide (100 μg/ml) and excluded from the measurements. Acquisitions were performed on a FACS Calibur flow cytometer (Becton-Dickinson Bioscience, Franklin Lakes, NJ, USA). Data analysis was conducted with FCS Express V2 software (version 2, De Novo Software, Los Angeles, CA, USA) following gating for the designated population.

### In vitro multipotent differentiation assays

The multi-lineage differentiation potential of human MSCs (n=3, P3) was analyzed by applying the standard procedures used by Pittenger *et al* (12). For adipogenesis, 2×10^5^ cells/cm^2^ were seeded. Five days after reaching confluence, the hBMMSCs were treated for three days with induction media consisting of α-MEM (Gibco Laboratories, Gaithersburg, MD, USA) supplemented with 10% FBS, 1.0×10^−7^ M dexamethasone (Sigma, St. Louis, MO, USA), 1.0×10^−9^ M insulin, 0.5 mM 1.0×10^−7^ M ascorbic acid and 7×10^−3^ M sodium β-glycerophosphate for 18 days at 37°C in a humidified atmosphere containing 5% CO_2_ and then for two days with maintenance media consisting of α-MEM, FBS and 1.0×10^−9^ M insulin. Sudan black B staining of the lipid droplets was performed as previously described (12). Sudan black B concentrations were calculated by comparison with a Sudan black B dye standard curve and expressed as nmol/ml following normalization against the total cellular protein, and is expressed as nmol/mg protein. The above experiments were performed at least three times independently and each experiment was conducted in quadruplicate.

For osteogenesis, hBMMSCs were seeded at a density of 2×10^5^ cells/cm^2^ and grown under osteogenic induction conditions in DMEM (Gibco Laboratories) supplemented with 1.0×10^−7^ M dexamethasone, 1.0×10^−7^ M ascorbic acid and 7×10^−3^ M β-glycerophosphate sodium for seven days at 37°C in a humidified atmosphere containing 5% CO_2_. The cells were collected following incubation with 0.25% trypsin-EDTA for 5 min. Following centrifugation, the cells were suspended in 100 ml normal saline and frozen and thawed three times at −200°C. The suspension was centrifuged at 250 × g for 10 min and osteocalcin contents were determined using the enzyme dynamics method.

### In vivo multipotent differentiation assays

For osteogenesis, five nude mice were inoculated subcutaneously with P15 hBMMSCs (2.5×10^7^) mixed with an equal volume of Matrigel^®^, and osteogenesis was observed as described previously (16). Myogenesis was studied as described by Caterson *et al* (16). For the controls, six nude mice were injected in the hind leg with absolute alcohol (n=3) or Dil-labeled hBMMSCs (2×10^6^) (n=3). For the treatment group, six nude mice were injected with absolute alcohol followed 2 h later by Dil-labeled hBMMSCs (2×10^6^). The tissue sections of leg muscles were prepared and incubated with the monoclonal antibody against myoglobin at day three and with the monoclonal antibody against MyoD1 at day 12 and were examined under a fluorescent microscope (CK40/BK51, Olympus Corporation, Tokyo, Japan).

### Hematopoietic reconstitution

The nude mice (6 to 8-week old males), were obtained from Shanghai Super-B&K laboratory animal Corp., Ltd (Shanghai, China). The mice were handled and housed according to protocols approved by the Shanghai Medical Experimental Animal Care Commission. A total of 4 h after the nude mice were sublethally (650 cGy) irradiated, each mouse was infused via the tail vein with 2×10^6^ hBMMSCs. In addition, 0.5 *μ*g granulocyte colony-stimulating factor was administered daily for three days. The control nude mice were infused with normal saline. The mice were sacrificed at different time-points for analysis. Peripheral blood samples were collected 48–144 h following infusion of hBMMSCs for leukocyte count and smear. The cells were also removed from bilateral femurs by flushing with PBS. The firmly adhered cells of the endosteal region were recovered by digesting the crushed bone with collagenase type IV (0.03%) and dispase (2 U/ml). The bone marrow cells were pooled in a tube and the erythrocytes were lysed by treatment with Grey’s solution. Mononuclear cells were collected and prepared for smears. The smears were then fixed in acetone and stained with PE-conjugated anti-human CD45 antibody and FITC-conjugated anti-human CD34 antibody, examined under a fluorescent microscope (Olympus Corporation) and the images were captured accordingly.

### RNA extraction and gene expression profiling

Total cellular RNA was extracted from hBMMSCs of P7 and P65 using the TRIzol reagent as instructed by the manufacturer (Invitrogen Life Technologies). RNA (1 μg; RIN value >6) was used for generation of second-strand cDNA, and cRNA was amplified with the Oligo dT primer, then biotinylated and fragmented with One-Cycle Target Labeling and control reagents (Affymetrix, Inc., Santa Clara, CA, USA), followed by hybridization to U133 Plus 2.0 array overnight (17 h) according to the manufacturer’s instructions. Finally, the hybridized DNA microarray was fluorescence stained with GeneChipFluidics Station 450 (Affymetrics) and scanned with a GeneChip Scanner 3000 (Affymetrics). GCOS1.2 Affymetrix GeneChip Command Console (TGCC) was employed to extract the data in the microarray followed by analysis with the Robust Multi-Array Average (RMA) method. The relative gene expression was calculated. A difference of >2 or <0.5*-*fold and reliability (P<0.05) was considered to indicate significant differences in gene expression.

## Results

### Morphological characteristics of cultured hBMMSCs

**Figure 1:**
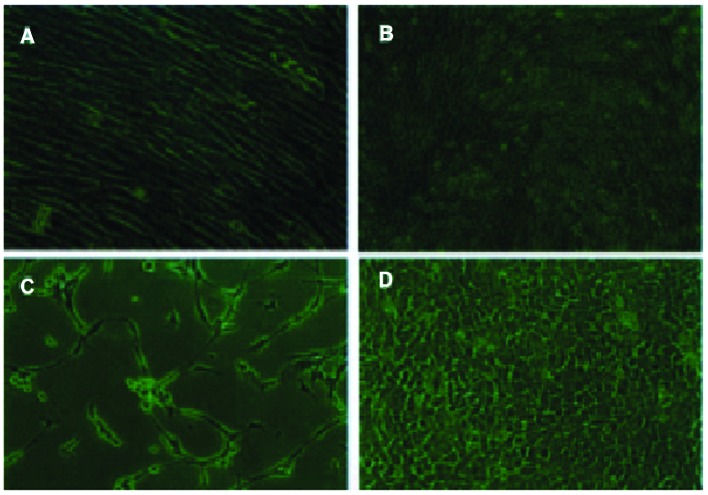
Morphological characteristics of human bone marrow mesenchymal stem cells. (A), ×200; (B), ×40; and (C) and (D), ×100.

**Figure 2:**
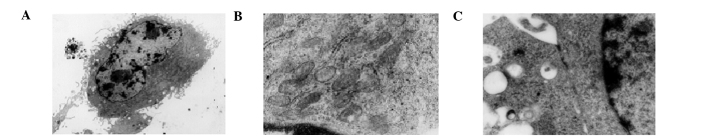
Morphological features of human bone marrow mesenchymal stem cells following one week of culture visualized by electron microscopy. (A) Nuclei were large and irregular in shape with 2 to 3 nucleoli and scant nuceloplasm. (B) The cytoplasm was rich in mitochondria and rough endoplasmic reticula. (C) Gap junctions.

**Figure 3:**
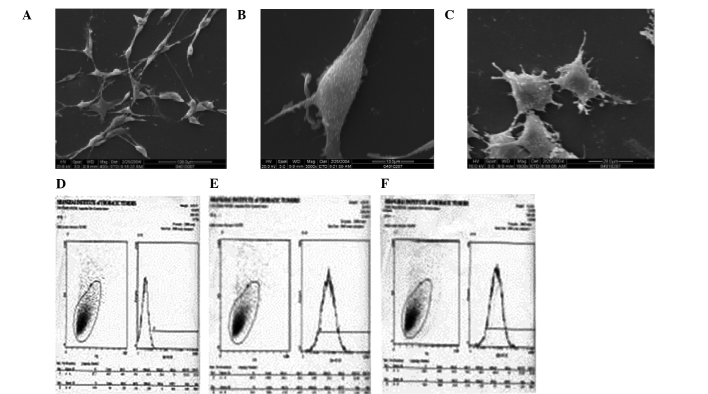
(A–C) Phenotypes of cultured hBMMSCs visualized by transmission electron microscopy. (A) hBMMSCs were elongated or polygonal; (B) short microvilli; (C) projections; (D–F) Flow cytometric analysis showed that hBMMSCs demonstrate pluripotent differentiation potential *in vitro*. hBMMSCs, human bone marrow mesenchymal stem cells.

The majority of hBMMSCs were elliptical following one week of culture with abundant cytoplasms and prominent nuclei. Light microscopy demonstrated that the cells resembled fibroblasts in shape and were arranged in parallel or whorls (Fig. 1A–D). Electron microscopy of cultured hBMMSCs revealed that the nuclei were large and irregular in shape with 2–3 nucleoli and scant nuceloplasm (Fig. 2A). The cytoplasms were rich in mitochondria and rough endoplasmic reticula (Fig. 2B) with gap junctions (Fig. 2C). Transmission electron microscopy further demonstrated that the cultured hBMMSCs were elongated or polygonal (Fig. 3A) with short microvilli (Fig. 3B) and projections (Fig. 3C). No apparent changes in cell morphology were observed for cells up to P10. The doubling time for P10, P20 and P40 hBMMSCs was 32, 36 and 32 h, respectively.

Flow cytometric studies revealed that the positive rate of CD31 was 31.1% in P4 hBMMSCs and 18.6% in P10 hBMMSCs. CD105 and CD106 were expressed in 99 and 95% of P25 hBMMSCs, respectively (Fig. 3D–F).

**Figure 4:**
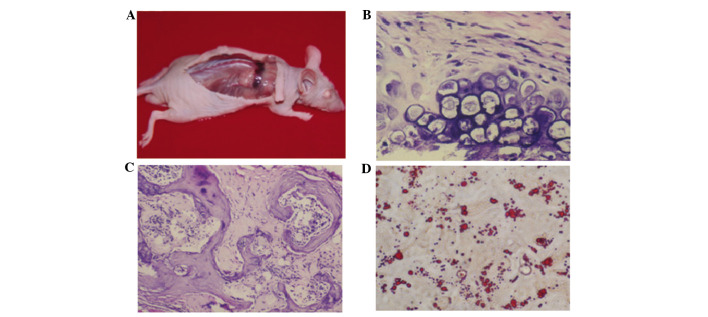
(A–D) hBMMSCs demonstrated pluripotent differentiation potential *in vitro* and *in vivo*. (A) *In vivo* osteogenesis test: Bone tissue formed subcutaneously on the back of the nude mice 35 days after hBMMSCs inoculation. (B) Histology results showed endochondral bone formation (hematoxylin and eosin staining, ×200) (C) Histology results showed simultaneous bone tissue formation (hematoxylin and eosin staining, ×100) (D) *In vitro* adipogenesis test: Sudan black B staining lipid droplets appeared at day 18 post 113 induction of adipogenesis. hBMMSCs, human bone marrow mesenchymal stem cells.

An *in vivo* osteogenesis test demonstrated subcutaneous bony tissue formation on the back of the nude mice 35 days post-hBMMSCs inoculation (Fig. 4A). Histology analysis showed a predominant endochondral bone formation process (Fig. 4B) in *in vivo* bone tissue formation (Fig. 4C). In adipogenesis, lipid droplets appeared at day 18 post induction of adipogenesis, which increased in size and occupied the entire cell (Fig. 4D). In osteogenesis, it was identified that the induced hBMMSCs were elongated, contained numerous particles and were positive for osteocalcin (Fig. 4B). The osteocalcin content was 12±0.5 mg/ml for induced P10 hBMMSCs and 12±0.5 mg/ml for induced P25 hBMMSCs, which was ~3× higher than that of the unstimulated cells. Furthermore, no expression of osteocalcin was observed in the induced P38 and P57 hBMMSCs.

**Figure 5:**
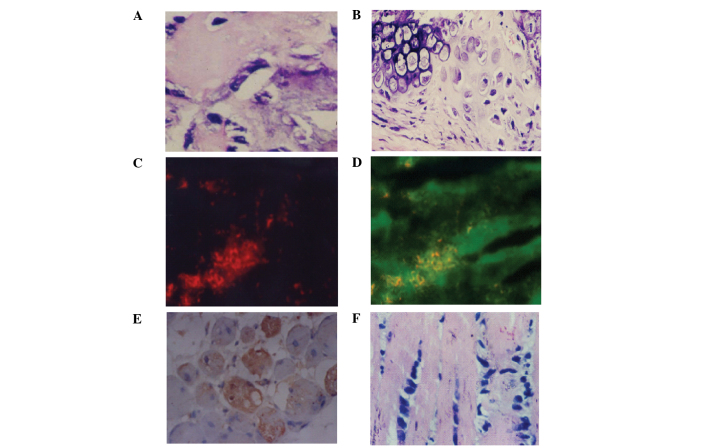
(A) Bone tissue formation. (B) Hematoxylin and eosin staining further revealed chondrocytes and the formation of bone tissues. Magnification, ×200. (C–F) Hematopoietic reconstitution by hBMMSCs. hBMMSCs, human bone marrow mesenchymal stem cells. Magnification, ×200.

For osteogenesis, palpable masses were detected from day 35 post inoculation of hBMMSCs, and bone tissue formation was observed (Fig. 5A). Hematoxylin and eosin (H&E) staining further revealed chondrocytes and the formation of bone tissues (Fig. 5B). For myogenesis, at day six after subcutaneous inoculation, hBMMSCs differentiated to myocytes and were positive for myoglobin (Fig. 5C and D). At day 12, H&E staining demonstrated striations in the cytoplasm of differentiated hBMMSCs at day 12 post inoculation (Fig. 5E) and the cells were positive for MyoD1 (Fig. 5F). The control nude mice revealed no formation of muscle cells and were negative for myoglobin and MyoD1.

**Figure 6:**
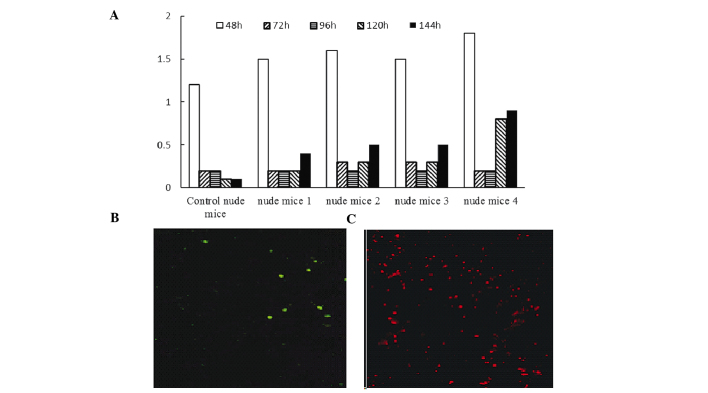
Gene expression profile changes in early and late hBMMSCs. (A) White blood cell counts in the peripheral blood (1×10^6^/dl); (B) CD34-positive cells were detected; magnification, ×100. (C) CD45-positive cells were detected. hBMMSCs, human bone marrow mesenchymal stem cells; magnification, ×100.

White blood cell counts in the peripheral blood of control mice irradiated at sublethal doses gradually decreased and did not recover to normal up to 144 h following irradiation (Fig. 6A). In irradiated nude mice reconstituted by hBMMSCs, the white blood cell count briefly dropped following irradiation but gradually recovered. CD45- and CD34-positive cells were not detected in the peripheral blood and bone marrow of the control nude mice. In irradiated nude mice reconstituted by hBMMSCs, CD45- and CD34-positive cells were detected 72 h and 144 h post induction (Fig. 6C–D).

The gene microarray analysis of P7 and P57 hBMMSCs demonstrated that 20 genes were upregulated >2*-*fold and 40 genes were downregulated >2*-*fold in the P57 hBMMSCs. The 10 most upregulated and 10 most downregulated genes are summarized in Table I.

## Discussion

In the present study, hBMMSCs were isolated from a surgical patient and stably expanded *ex vivo* until P65. Flow cytometric analysis of P25 hBMMSCs demonstrated that the cells expressed CD105 and CD106 but not CD14, CD34 and CD45, and exhibited apparent contact inhibition. Furthermore, these cells were able to form cartilage and osteoid tissues in nude mice. In addition, bone marrow cells were observed in the osteoid tissue and formation of erythrocytes was also noted, suggesting there was ongoing erythropoiesis in the osteoid tissue. Myogenesis in nude mice was also observed following inoculation of hBMMSCs. H&E staining revealed typical striations in the newly formed skeletal muscles. These findings indicated that hBMMSCs are able to differentiate into blood and muscle cells. Early infusion of hBMMSCs allows hematopoiesis in the early stage of bone marrow failure, which provides time for recovery of the bone marrow from the infusion of autologous hematopoietic stem cells. This is consistent with the microenvironment induction theory, stating that stem cells form tissue-specific cells depending on the microenvironment (18).

The gene expression profiles of P7 and P57 hBMMSCs were investigated and it was identified that 20 genes were upregulated >2 fold and 40 genes were downregulated >2 fold. These genes are involved in angiogenesis, cell cycle control and the regulation of proliferation and apoptosis. Furthermore, the late passages of hBMMSCs were associated with ploidy compared with the early passages of hBMMSCs (data not presented). These findings indicated that the passage of hBMMSCs may be associated with gene mutations and gene expression changes. Therefore, early passages of hBMMSCs should be used for stem cell therapy. These findings are consistent with those by Maitra *et al* (17).

Mesenchymal stem cells possess pluripotent potential and are induced to differentiate into cells of various types for organ regeneration, including osteoblasts, chondrocytes, cardiomyocytes, myocytes, adipocytes and hematopoietic cells. As a result, they hold significant potential for broad clinical applications in the treatment of numerous malignancies. The present study found that it is feasible to isolate and long-term culture hBMMSCs from patients, and that it is safe to use hBMMSCs within 30 passages. An hBMMSCs cell line was established which may be used for further studying the biological properties of hBMMSCs for tissue engineering.

## Figures and Tables

**Table I:** Ten most upregulated and downregulated genes in cultured late human bone marrow mesenchymal stem cells.

A, 10 most upregulated genes			

Probe set ID	Gene symbol	Chromosomal location	HBHSC-60 vs HBmSc-Signal log ratio
201242_s_at	ATP1B1	chr1q24	2.4
219567_s_at	FLJ21144	chr1p34.2	2.3
241940_at	---	---	2.2
237566_at	---	---	2.1
204298_s_at	LOX	chr5q23.2	1.8
209427_at	SMTN	chr22q12.2	1.7
219778_at	ZFPM2	chr8q23	1.6
204881_s_at	UGCG	chr9q31	1.5
209283_at	CRYAB	chr11q22.3-q23.1	1.5
201110_s_at	THBS1	chr15q15	1.5

B, 10 most downregulated genes			

Probe set ID	Gene symbol	Chromosomal location	HBHSC-60 vs HBmSc-Signal log ratio

220822_at	---	---	−3.5
226677_at	ZNF521	chr18q11.2	−3.5
202227_s_at	BRD8	chr5q31	−3.2
230030_at	HS6ST2	chrxq26.2	−2.9
202213_s_at	CUL4B	chrxq23	−2.8
208612_at	GRP58	chr15q15	−2.5
201444_s_at	ATP6AP2	chrxq21	−1.9
217572_at	---	---	−1.9
201310_s_at	C5orf13	chr5q22.1	−1.8
205350_at	CRABP1	chr15q24	−1.8
